# Characterization of the mitochondrial genome of the MAX1 type of cytoplasmic male-sterile sunflower

**DOI:** 10.1186/s12870-019-1637-x

**Published:** 2019-02-15

**Authors:** Maksim S. Makarenko, Alexander V. Usatov, Tatiana V. Tatarinova, Kirill V. Azarin, Maria D. Logacheva, Vera A. Gavrilova, Renate Horn

**Affiliations:** 1 Southern Federal University, Rostov-on-Don, Russia; 2 University of La Verne, La Verne, CA USA; 3 Institute for Information Transmission Problems, Moscow, Russia; 4 Institute for General Genetics, Moscow, Russia; 5 Siberian Federal University, Krasnoyarsk, Russia; 6 Skolkovo Institute of Science and Technology, Moscow, Russia; 7 The N.I. Vavilov All Russian Institute of Plant Genetic Resources, Saint Petersburg, Russia; 8 University of Rostock, Institute of Biological Sciences, Plant Genetics, Rostock, Germany

**Keywords:** Cytoplasmic male sterility, Sunflower, Mitochondrial genome rearrangements, CMS MAX1, *atp6* chimeric gene

## Abstract

**Background:**

More than 70 cytoplasmic male sterility (CMS) types have been identified in *Helianthus*, but only for less than half of them, research of mitochondrial organization has been conducted. Moreover, complete mitochondrion sequences have only been published for two CMS sources – PET1 and PET2. It has been demonstrated that other sunflower CMS sources like MAX1, significantly differ from the PET1 and PET2 types. However, possible molecular causes for the CMS induction by MAX1 have not yet been proposed. In the present study, we have investigated structural changes in the mitochondrial genome of HA89 (MAX1) CMS sunflower line in comparison to the fertile mitochondrial genome.

**Results:**

Eight significant major reorganization events have been determined in HA89 (MAX1) mtDNA: one 110 kb inverted region, four deletions of 439 bp, 978 bp, 3183 bp and 14,296 bp, respectively, and three insertions of 1999 bp, 5272 bp and 6583 bp. The rearrangements have led to functional changes in the mitochondrial genome of HA89 (MAX1) resulting in the complete elimination of *orf777* and the appearance of new ORFs - *orf306, orf480, orf645* and *orf1287*. Aligning the mtDNA of the CMS sources PET1 and PET2 with MAX1 we found some common reorganization features in their mitochondrial genome sequences.

**Conclusion:**

The new open reading frame *orf1287*, representing a chimeric *atp6* gene, may play a key role in MAX1 CMS phenotype formation in sunflower, while the contribution of other mitochondrial reorganizations seems to appear negligible for the CMS development.

**Electronic supplementary material:**

The online version of this article (10.1186/s12870-019-1637-x) contains supplementary material, which is available to authorized users.

## Background

Widespread use of hybrid seed production for cross-pollinated crop plants makes cytoplasmic male sterility (CMS) one of the most desired agricultural traits [1]. More than 70 cytoplasmic male sterility types have been identified in *Helianthus* [2], but almost all sunflower hybrids are currently based on a single source of CMS – PET1, discovered 50 years ago by Leclercq [3] in an interspecific hybrid between *Helianthus petiolaris* Nutt and the cultivated sunflower - *H. annuus* L. Such genetic homogeneity can result in decrease of yield and disease resistance [4, 5]. However, potential new sources of CMS (CMS cytotypes) have not been sufficiently investigated at the molecular level, presenting a serious obstacle for their introduction into commercial hybrid breeding [6].

The CMS phenomenon provides a convenient model to study nuclear-cytoplasmic interaction and its role in pollen development [7–9]. Nuclear male sterility is controlled only by nuclear genes, whereas cytoplasmic sterility occurs as a result of the interaction of a male sterile cytoplasm with nuclear sterility factors [10, 11]. Nuclear control of CMS is accomplished by *Rf* genes performing fertility restoration [12].

Genetic basis of cytoplasmic male sterility is formed by structural rearrangements of mitochondrial DNA (mtDNA) [12–14]. New chimeric ORFs in the mtDNA of CMS lines are formed from fusion of known functional mitochondrial genes with uncharacteristic DNA sequences [1, 12, 15]. For instance, a new open reading frame (*orfH522*) appeared in the 3′-flanking region of the *atp1* gene encoding the alpha subunit of mitochondrial F1 ATPase in the CMS PET1 of sunflower as a result of an 11-kb-inversion and a 5-kb-insertion [16, 17]. This *orfH522* is co-transcribed with the *atp1* gene as a polycistronic mRNA [18] and translated into the CMS-specific 16-kDa-protein [19]. Molecular basis of another CMS type of sunflower - PET2 - has been studied recently [20, 21]. Both CMS types (PET1, PET2) were obtained from interspecific crosses between *H. petiolaris* and the cultivated sunflower *H. annuus* [2, 22]. However, structural rearrangements of the PET2 mtDNA leading to the emergence of new open reading frames differ from those in CMS-PET1. Multiple rearrangements have been detected in the mitochondrial sequence of PET2: two translocations (27.5 kb and 106.5 kb), two deletions (711 bp and 3780 bp), as well as insertions of 5050 bp and 15,885 bp [20]. Some of these mitochondrial genome reorganizations resulted in formation of novel ORFs (*orf645*, *orf2565, orf228,* and *orf285*) with transcriptional activity specific to the CMS PET2. It was suggested that *orf228* (*orf231* including the stop codon) and *orf285* (*orf288* including the stop codon)*,* being chimeric *atp9* ORFs, may be the main driver for the development of PET2 CMS phenotype [20, 21]. Reduction of the co-transcript of the two ORFs could be specifically detected in the anthers of fertility-restored hybrids, supporting their role in the CMS phenotype [21].

Except for PET1 and PET2, the other CMS types of sunflower are poorly investigated. To the best of our knowledge, only one comparative study of mitochondrial genomes organizations including 28 CMS types of sunflower has been conducted [23]. There were also several independent attempts to analyze the mtDNA of MAX1 [24, 25] and PEF1 [26, 27] CMS types. The investigations demonstrated that MAX1 CMS source significantly differs from the PET1 and PET2 mitotypes [23–25]. Thus MAX1 CMS source, which was initially obtained by the interspecific hybridization of an annual sunflower (*Helianthus annuus* L.) with the perennial species *Helianthus maximiliani* Schrad [28], is insufficiently explored and is of particular interest.

In the present study, we have investigated structural changes in the mitochondrial genome of HA89 (MAX1) CMS sunflower line and identified possible causes for the development of male sterility in the MAX1 mitotype.

## Materials and methods

### Plant material

The CMS line HA 89 (MAX1) as well as the fertile line HA 89 with the normal cytoplasm were obtained from the genetic collection of the N. I. Vavilov Institute of Plant Genetic Resources (VIR, Russia). Sunflower plants were grown in regularly irrigated pots in the growth chamber KBWF 720 (Binder, Germany) at the following growth conditions: 26 °C (78.8 F) temperature, 70% humidity, and 10/14 h dark/light cycle. For the DNA and RNA manipulations, we used leaf tissue from five plants, which were on the same growth stage and had maximal phenotypic similarity.

### Mitochondrial DNA extraction, genome library construction, and NGS sequencing

We extracted the organelle fraction with a reduced amount of nuclear DNA from leaves of 14-days-old sunflower seedlings as described by Makarenko et al. [29]. DNA isolation was performed with the PhytoSorb kit (Syntol, Russia), according to the manufacturer’s protocol. The NGS libraries were prepared with Nextera XT DNA Library Prep Kit (Illumina, USA), following the sample preparation protocol by Illumina. The concentration of the prepared library was measured with the Qubit fluorometer (Invitrogen, USA) and qPCR. Fragment length distribution was determined with Bioanalyzer 2100 (Agilent, USA). Libraries for NGS sequencing were diluted up to the concentration of 10 pM and sequenced on NextSeq 500 sequencer using High Output v2 kit (Illumina, USA). A total number of 10,174,497,150-bp paired reads were generated.

### Mitogenome assembly and annotation

Quality of reads was determined with FastQC (https://www.bioinformatics.babraham.ac.uk/projects/fastqc/). Trimming of adapter-derived and low quality (Q-score below 25) reads was performed with the Trimmomatic software [30]. The whole genome assembly was done using the SPAdes genome assembler v 3.10.1 [31]. High coverage (depths > 100) contigs were then manually assembled based on the scaffolds and presence of bridge contigs. Validation was performed by mapping the initial reads on the assembled genome sequence using bowtie 2 [32]. The assembled genome was aligned to the reference sunflower mitochondrial genome (NC_023337.1) using BLAST. Verification of the discovered rearrangements was made by PCR. Primer sequences are presented in Additional file 1. The mitochondrial genome was annotated with glimmer 3.02b [33] and BLAST tools. Potential ORFs were identified using ORFfinder (https://www.ncbi.nlm.nih.gov/orffinder). Graphical genome map was generated using the OGDRAW tool [34]. Transmembrane domains were predicted using the TMHMM server v.2.0 (available online: http://www.cbs.dtu.dk/services/TMHMM-2.0/). We have conducted analysis of distribution of transcription factor binding sites (TFBS) using Match [35] and cisExpress [36, 37]. The complete mitochondrion sequence of CMS line HA89(MAX1) has been deposited to GenBank under the accession number MH704580.1.

### Reference genome

Comparison of HA89 (MAX1) was with HA 89 fertile line genome (available at 10.6084/m9.figshare.7265648.v1); this sequence will later be deposited to NCBI GenBank. In our analysis we used positions of a publicly available mitochondrial genome of a fertile line that differs from HA89 by two nucleotides - HA412 (NC_023337.1).

### RNA extraction and qRT-PCR

Total RNA from leaves was extracted with guanidinium thiocyanate-phenol-chloroform reagent kit – ExtractRNA (Evrogen, Russia). RNA quality and concentration were measured using the NanoDrop 2000 spectrophotometer (Thermo Fisher Scientific, USA) and the Qubit fluorometer (Invitrogen, USA). 0.5 μg of total RNA was treated with DNAse I (Thermo Fisher Scientific, USA) per manufacturer’s instruction. The cDNA was synthesized using random primers and MMLV RT kit (Evrogen, Russia). The qPCR was performed with PCR kit with EvaGreen dye (Syntol, Russia) on Rotor-Gene 6000 (Corbett Research, Australia). Primer sequences are presented in Additional file 1.

## Results

**Fig. 1:**
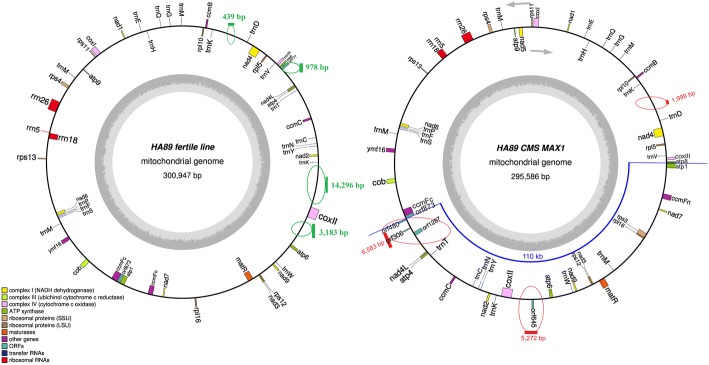
Graphical mitochondrial genome maps of the HA89 fertile and HA89 (MAX1) CMS lines. Arrows denote the genes transcription orientation. Green bars denote the locations of revealed larger deletions and the sizes. Red bars denote the locations of revealed larger insertions and the sizes. The blue line indicates the 110 kb inverted region

We assembled the complete mitochondrial genome of HA89 sunflower line with MAX1 cytoplasmic sterility type. The master chromosome of HA89 (MAX1) consists of 295,586 bp (Fig. 1). HA89 (MAX1) mitochondrial genome is 5361 bp shorter than the mtDNA genome of HA89 fertile analogue. The HA89 (MAX1) mitochondrial genome showed several rearrangements as compared to the HA89 fertile genome. We have identified eight significant reorganization events of HA89 (MAX1) mtDNA: one large inverted region, four deletions, and three insertions.

Comparative analysis of HA89 (MAX1) and its fertile analogue identified one inverted region, which is approximately 110 kbp long. This rearrangement includes a region flanked by *orf873* and *atp8* and is highlighted in Fig. 1. We like to emphasize that this rearrangement covers more than 30% of the entire mitochondrial genome. Presence of this huge rearrangement is also associated with most of the identified deletions and insertions. We have found four long deletions (more than 100 bp) in HA89 (MAX1) as compared with the HA89 fertile line: 439 bp, 978 bp, 3183 bp and 14,296 bp. All deletions except for the smallest one (439 bp) are associated with this 110 kb region. The 978 bp deletion is flanking the 3’region of the MAX1 specific 110 kb rearrangement. Matching this deletion to the sunflower fertile line mitochondrial genome map, we determined that it was located between the genes *nad4L* and *atp8* (35,559–36,537 positions in the HA89 fertile mitochondrion). This area of the mitochondrial genome normally codes for *orf777.* Thus the 978 bp deletion results in the absence of *orf777* in the HA89 (MAX1) mitochondrial genome. The other two deletions (3183 and 14,296 bp) also occurred within this 110 kb region of the MAX1 mitochondrial genome. Compared to the HA89 fertile line, both deletions are in intergenic regions and do not directly impact coding sequences. The 3183 bp deletion is localized between *atp6-coxII* (272,051–275,233 positions in the HA89 fertile mtDNA). The 14,296 bp deletion predominantly affects the large repeat region of the sunflower mtDNA - *coxII-nad2* (286,492–300,787 positions in the HA89 fertile mitochondrion). The smallest deletion (439 bp) also belongs to a large repeat intergenic region of mtDNA – *nad4-ccmB* (56,166–56,604 positions in the HA89 fertile mtDNA).

**Fig. 2:**
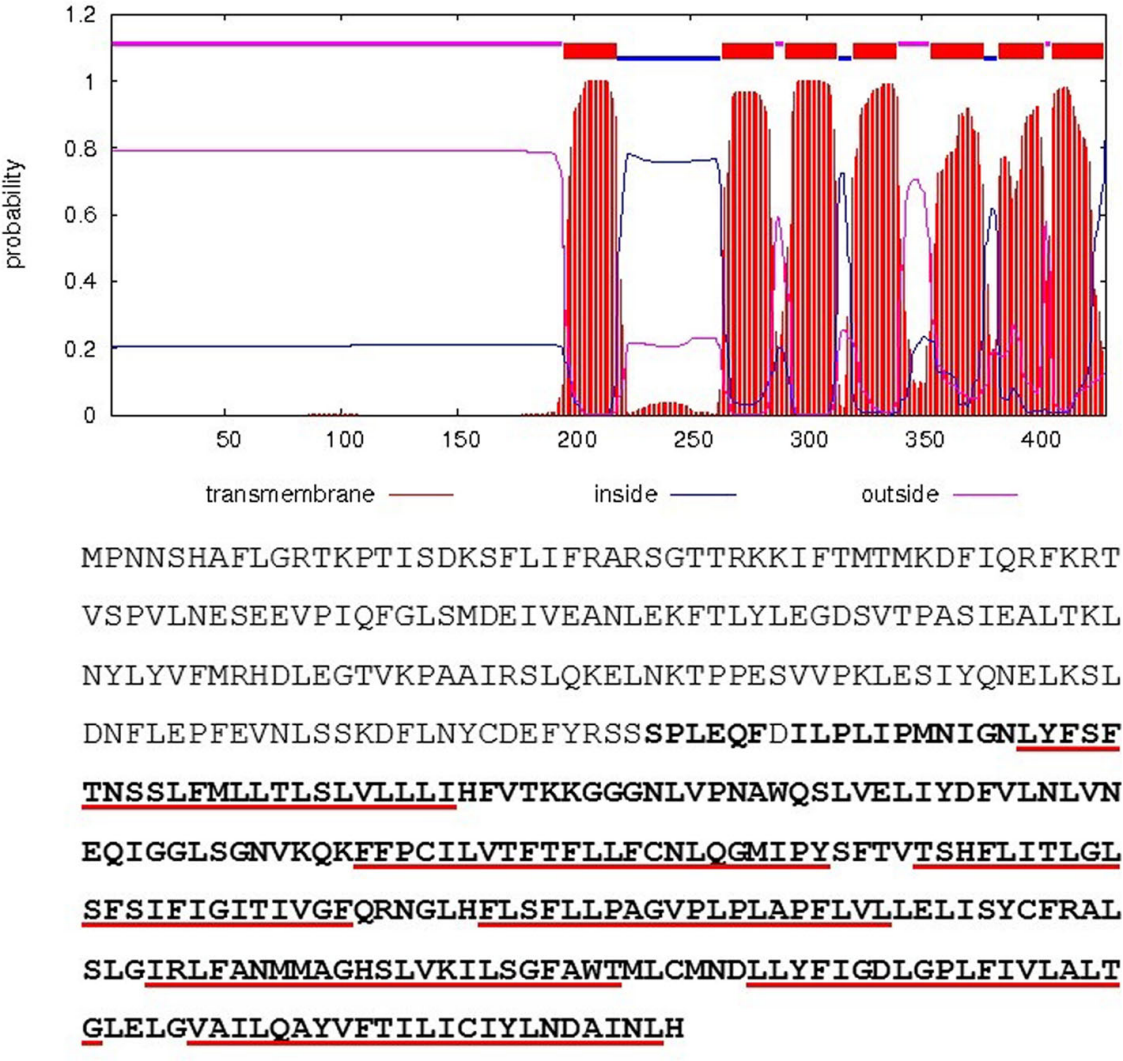
Comparison of orf1287 and atp6 encoded proteins and prediction of transmembrane helices. Identical amino acids are in bold. Transmembrane domains as predicted by TMHMM are marked by red bars

We have also detected several insertions in the HA89 (MAX1) CMS line. Three long (> 100 bp) insertions were found with sizes of 1999, 5272 and 6583 bp, respectively. The smallest one (1999 bp) is in the intergenic region *nad4-ccmB*, co-located with the 439 bp deletion. Thus, the occurrence of the 1999 bp insertion and the deletion of the mtDNA large repeat region (14,296 bp deletion) could be connected. On one hand, insertion in one copy of mtDNA repeat region could impair recombination of mtDNA molecules and eventually lead to the elimination of the other repeat copy in the mitochondrial genome. On the other hand, initial deletion of one copy of the repeat region can make the remaining copy more prone to the structural changes. The 1999 bp insertion has 100% similarity to the inverted repeat region of the sunflower chloroplast DNA. This insertion does not lead to the formation of new open reading frames; however, it includes a part of the *rrn23* gene. The 5272 bp insertion is co-localized with the other deletion site (3183 bp) - *atp6-coxII* and makes up a part of the rearrangement encompassing the 110 kb inverted region. The 5272 bp insertion results in a single large (more than 300 bp) new open reading frame – *orf645*. We detected transcriptional activity for *orf645* in MAX1 CMS line by RT-qPCR. The largest insertion (6583 bp) identified in the HA89 (MAX1) genome is localized in the 5′ flanking region of the 110 kb rearrangement between *orf873* and *nad4L* (Fig. 1). The 6583 bp insertion includes 1233 bp 99% identical to another part of the mitochondrial genome (205,493–206,725 positions of MAX1 mtDNA), creating a duplication of this mitochondrial region. The duplicated region includes a partial sequence of the *atp6* gene. The 6583 bp insertion results in the chimeric open reading frame *orf1287*. The *orf1287* is a fusion of 530 bp (in the 5′-end) of an unknown sequence with 760 bp (in the 3′-end) identical to *atp6*. This *orf1287* would encode an elongated protein of 429 amino acids with a partial homology to ATP6. The chimeric *atp6* transcript of the *orf1287* was detected in the male sterile HA89(MAX1) line by qPCR, but not in the fertile line. In addition to the *orf1287,* we predicted in the 6583 bp insertion two more potential ORFs - *orf306* and *orf480.* The search for transmembrane domains in these ORFs showed no transmembrane helix in *orf306,* but one possible membrane related domain in *orf480* (prediction chance was about 50%) and seven transmembrane helices in *orf1287* (Fig. 2).

The analysis of transcription factor binding sites (TFBS) revealed significant differences in distribution of TBFS in the promoter region of *atp8*. HA89 (MAX1) CMS promoter is 166 bp long, whereas HA 89 fertile line promoter is considerably longer, 384 bp. Earlier studies in radish had shown that in the male sterile mitochondrial genome the promoter of the *atp8* gene was lost [38]. The same study demonstrated that in CMS radish, *atp8* was co-transcribed with *orf138* and *trnfM*, but transcribed from its own promoter in a fertile variety. In the CMS MAX1 mitochondrial genome the genes *atp1*, *atp8* and *coxIII* are located next to each other (Fig. 1), hinting at a possibility of co-transcription using the promoter of *atp1*. The promoter sequences of *atp1* are not conserved between HA 89 (MAX1) and HA 89 fertile lines.

## Discussion

Although more than 70 types of cytoplasmic male sterility have been identified in sunflower [2], but research on their mitochondrial organization has been conducted only for less than half of them [23–26, 39, 40]. Assembly of plant mitochondrial genomes is always a difficult task [41]. It is especially hard to assemble a single mitochondrial DNA molecule, the so-called “master chromosome”. Most problems are associated with dynamic rates of rearrangements in plant mitochondrial genomes, resulting in the formation of a variety of subgenomic forms [42, 43]. Complete mitochondrial sequences have been investigated only for two CMS sources in sunflower [20]. Comparing the current research data with our previous study [20], where we analyzed mtDNA of PET1 (MG735191.1) and PET2 (MG770607.2) CMS types of sunflower, we can formulate a hypothesis about the molecular basis of CMS.

Matching the mtDNA of PET1 and MAX1 CMS sources, we found common mitochondrial rearrangement features. Both lines have a similar “rearrangement hot spot” near the *orf873* gene. A part of the 6583 bp insertion (MAX1) is complimentary (with 99% identity) to the 4732 bp insertion (PET1). Such sequence homology leads to the presence of *orf306* in both lines and *orf480* with partial similarity to *orfH522* in MAX1. Comparison of PET2 and MAX1 mtDNA also showed similarities in reorganizations of their mitochondrial genomes. Both CMS types have deletions in the *nad4L-atp8* region and are characterized by the total elimination of *orf777*. About 80% of the nucleotide sequence of 5272 bp insertion (MAX1) is reverse-complement (with 99% identity) to the 5050 bp insertion (PET2). Moreover, *orf645* is found in both CMS lines. The 6583 bp insertion (MAX1) has similarity to the 15,885 bp insertion in PET2. Both insertions have a common region counting about 500 nucleotides and the 1233 bp duplication site (MAX1) is also identical in the PET2 mitochondrial genome. The provided data in the current study confirm the similarity of MAX1 and PET2 mtDNAs organization, which had been observed in former hybridization results [23]. However, the fertility restoration patterns for three CMS types (MAX1, PET1, PET2) differed considerably. For CMS MAX1 all investigated maintainer and restorer lines of PET1, apart from HA 89, restored fertility. But only two maintainers and one restorer line of CMS PET1 were able to restore the fertility of plants with PET2 CMS [44]. It indicates a different origin of male sterility in these three CMS types, which is confirmed by the molecular comparison of the CMS types presented in the current research.

CMS phenotype formation predominantly involves the appearance of new open reading frames with partial complementary to mitochondrial genes: ATPase complex subunits, respiratory-related proteins, etc. [12, 45]. Mitochondrial DNA deletions usually lead to male sterility, when they result in the creation of new ORFs or the impairing coding sequence of vital mitochondrial proteins [12]. Elimination of the entire *orf777*, encoding a protein with an unknown function, is unlikely to result in male sterility, despite the absence of the *orf777* in both CMS types of sunflower (PET2 and MAX1). Most CMS associated genes encode polypeptides with transmembrane domains [11, 46]. Among the discovered new ORFs are *orf306* and *orf645,* encoding proteins lacking transmembrane regions; therefore, it is unlikely that they might cause CMS by disturbing membrane integrity. The appearance of *orf645* in two CMS types (PET2 and MAX1) of sunflower is probably not accidental, and *orf645* could also be a conserved ORF in mitochondrial genomes of other *Helianthus* genus species. However, *orf480* has a higher likelihood to result in the appearance of the CMS phenotype. The *orf480* translated protein has one membrane related region and shows similarity to the ORFH522 protein*,* which plays a major role in formation of the PET1 CMS type [16]. However, in the case of the ORFH522 protein, there is a definite transmembrane helix that is formed by the N-terminal 22 amino acids (20–42 aa). In turn, the *orf480* translated polypeptide has only a membrane related region (predominantly inside the membrane’s layer) in the N-terminus (5–27 aa) and is unlikely to form a transmembrane helix. This *orf480*, also called *orfM160* in a prior MAX1 study [24], was not transcribed and was also present in the male fertile *H. maximiliani* population MAX30. That is why we also do not consider *orf480* to be the leading cause of the MAX1 CMS type in sunflower.

The chimeric *orf1287* identified in the HA89 (MAX1) mitochondrial genome, is a potential candidate gene for the CMS development. Neither the HA89 fertile line nor the CMS lines HA89 (PET1), HA89 (PET2) has mitochondrial genes homologous to *orf1287*. The *orf1287* encodes the *atp6* chimeric protein, which has 250 aa common to ATP synthase Fo subunit 6 C-terminus but has an additional extended and dissimilar N-terminal part (Fig. 2). All seven transmembrane helices present in the C-terminus of a normal ATP6 protein also exist in the protein encoded by *orf1287*. We searched for homologous proteins in the NCBI database and found a significant identity (more than 70%) to ATP synthase subunit 6 of sunflower line with another CMS type - ANT1 (CAA57790.1). Compared with a normal ATP synthase subunit 6 (351 aa long) in both types of CMS (MAX1, ANT1) the elongated proteins would be translated into proteins of 429 aa (MAX1) and 437 aa (ANT1), respectively. It should be noted that they have identical C-termini and highly similar N-termini. The *atp6* gene is one of the most frequently rearranged mitochondrial genes in plants [12, 47, 48]. Correlation between the chimeric *atp6* gene and CMS phenotypes have been found in several species, including pepper [48], maize [49], radish [50], monkeyflower [51], etc. It is interesting to note, that in sugar beet the chimeric ORF presented by the *atp6* 5′ elongated transcript has been translated in vivo and caused CMS phenotype [47]. From these observations, we suggest that the predicted translation product of *orf1287* may play an important role in MAX1 CMS type formation in sunflower.

## Conclusions

Eight significant major reorganization events of HA89 (MAX1) mtDNA have been determined: one 110 kb encompassing inverted region, four deletions – 439 bp, 978 bp, 3183 bp and 14,296 bp, and three insertions - 1999 bp, 5272 bp and 6583 bp. The rearrangements have led to functional changes in mitochondrial genome of HA89 (MAX1): complete elimination of *orf777* and appearance of new ORFs – *orf306, orf480, orf645* and *orf1287*. The predicted translation product of *orf1287* may play a key role in the development of the male sterile phenotype in CMS MAX1 in sunflower, while the contributions of other mtDNA reorganizations to the development of CMS phenotype appear to be unlikely.

## Additional file

Additional file 1: The primers sets used for HA89 (MAX1) genome reorganizations validation and gene expression analysis. *- has been also used in previous study (Makarenko et al., 2018). (DOCX 13 kb)
