# Supplementary material for: Characterization of the mitochondrial genome of the MAX1 type of cytoplasmic male-sterile sunflower
Source: BMC Plant Biol. 2019 Feb 15;19(Suppl 1):51. doi: 10.1186/s12870-019-1637-x (PMC6394147; doi:10.1186/s12870-019-1637-x)
Supplement: Supplementary file 1 — The primers sets used for HA89 (MAX1) genome reorganizations validation and gene expression analysis. *- has been also used in previous study (Makarenko et al., 2018). (DOCX 13 kb) [file 12870_2019_1637_MOESM1_ESM.docx]

| The purpose | Primer name | Primer sequence (5’-3’) | Line | The amplicon size (kbp) |
| --- | --- | --- | --- | --- |
| The 110 kbp rearrangement detection | atp1R* | CAGAAACGCTCAACTGTGGC | MAX1 | 1.5 |
|  | 37192R* | CAAGTGATCCCCCATCCAGG | Fertile | - |
|  | 200953F | GTAGTCGTGACCAACAGCCA | MAX1 | 2.05 |
|  | orf480R | GTGCAAACCCTCACGCAAG | Fertile | - |
| The 14,296 bp (the large repeat region) deletion detection | 141R | GAAGCCCTTTCCTTACTCCTCA | MAX1 | 2.5 |
|  | 284313F | GCTCGGCTAAATAGGCTCAAT | Fertile | 16.8 |
| The detection of 439 bp deletion and 1,999 bp insertion | 51542F | GCTGTACACCTGGGATTGTAG | MAX1 | 7.7 |
|  | 57735R-  294143R | GGGACGAGCTGCATCCTATT | Fertile | 6.2 |
| The detection of 3,183 bp deletion and 5,272 bp insertion | 270988F | GAGGTGGAGGGAAGCTAGGA | MAX1 | 6.6 |
|  | 275503R | TAACCGCTGCAAGAGTGAGG | Fertile | 4.5 |
| The analysis of *orf306* expression | orf306F | AAGAAAGGCACCTCTGGACG | MAX1 | 0.146 |
|  | orf306R | TCCGGGGGAAAGAAATCCAT | Fertile | - |
| The analysis of *orf480* expression | orf480F | GGGCGATGACCCGGATAAG | MAX1 | 0.136 |
|  | orf480R | -\|\|- | Fertile | - |
| The analysis of *orf*645 expression | orf645MAX | ACCCGGAGTTCTGTATCAGC | MAX1 | 0.134 |
|  | orf645F* | GCCTTCCACCTCTCGTTTGA | Fertile | - |
| The analysis of *orf1287* expression | orf1287F | CGGATTCTGGGGGTGTCTTG | MAX1 | 0.14 |
|  | orf1287R | TGTTACCCCCGCAAGTATCG | Fertile | - |
| The analysis of *atp1* expression | atp1F* | CCCATGGCACAGCCAGAATA | MAX1 | 0.14 |
|  | atp1R* | -\|\|- | Fertile | 0.14 |
| The analysis of *atp6* expression | atp6F* | AGAACTGTAACTGACAACGC | MAX1 | 0.106 |
|  | atp6R | ACCTGAGTCCGAGTCTGCATC | Fertile | 0.106 |

Supplementary Table 1. The primers sets used for HA89 (MAX1) genome reorganizations validation and gene expression analysis.

*- has been also used in previous study (Makarenko et al., 2018)
